# The Alternative Route to Heme in the Methanogenic Archaeon *Methanosarcina barkeri*

**DOI:** 10.1155/2014/327637

**Published:** 2014-01-23

**Authors:** Melanie Kühner, Kristin Haufschildt, Alexander Neumann, Sonja Storbeck, Judith Streif, Gunhild Layer

**Affiliations:** ^1^Institut für Mikrobiologie, Technische Universität Braunschweig, Spielmannstr 7, 38106 Braunschweig, Germany; ^2^Institut für Organische Chemie, Technische Universität Braunschweig, Hagenring 30, 38106 Braunschweig, Germany

## Abstract

In living organisms heme is formed from the common precursor uroporphyrinogen III by either one of two substantially different pathways. In contrast to eukaryotes and most bacteria which employ the so-called “classical” heme biosynthesis pathway, the archaea use an alternative route. In this pathway, heme is formed from uroporphyrinogen III via the intermediates precorrin-2, sirohydrochlorin, siroheme, 12,18-didecarboxysiroheme, and iron-coproporphyrin III. In this study the heme biosynthesis proteins AhbAB, AhbC, and AhbD from *Methanosarcina barkeri* were functionally characterized. Using an *in vivo* enzyme activity assay it was shown that AhbA and AhbB (Mbar_A1459 and Mbar_A1460) together catalyze the conversion of siroheme into 12,18-didecarboxysiroheme. The two proteins form a heterodimeric complex which might be subject to feedback regulation by the pathway end-product heme. Further, AhbC (Mbar_A1793) was shown to catalyze the formation of iron-coproporphyrin III *in vivo*. Finally, recombinant AhbD (Mbar_A1458) was produced in *E. coli* and purified indicating that this protein most likely contains two [4Fe-4S] clusters. Using an *in vitro* enzyme activity assay it was demonstrated that AhbD catalyzes the conversion of iron-coproporphyrin III into heme.

## 1. Introduction

Heme serves as an essential prosthetic group in many enzymes involved in fundamental biological processes in almost all organisms [1]. In eukaryotes and most bacteria, the biosynthesis of heme proceeds via a conserved, well characterized “classical” pathway [2]. In contrast, heme is formed via an alternative route in the archaea and some bacteria such as the sulfate-reducing *Desulfovibrio* species [3–9]. In both the classical and the alternative pathway, 5-aminolevulinic acid (ALA) serves as the common precursor for heme formation which is converted into the biosynthetic intermediate uroporphyrinogen III (UROGEN) in three consecutive enzymatic steps. In the classical pathway UROGEN is then converted into heme via the intermediates coproporphyrinogen III, protoporphyrinogen IX, and protoporphyrin IX [2].

**Figure 1:**
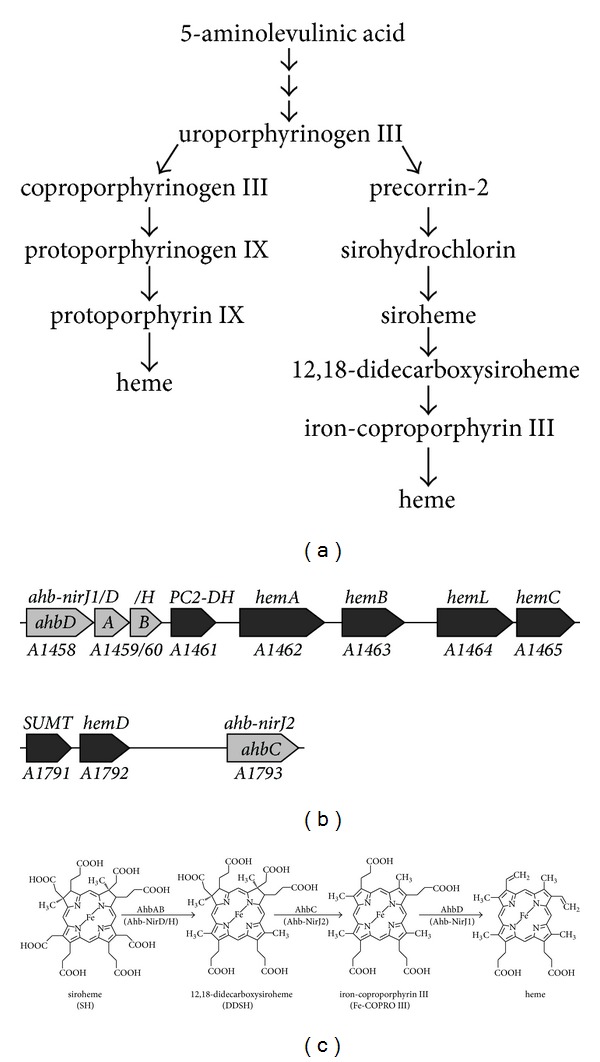
The alternative heme biosynthesis pathway in *Methanosarcina barkeri*. (a) Comparison of the classical heme biosynthesis pathway (left hand side) with the alternative pathway (right hand side). In both pathways heme is derived from the common precursor uroporphyrinogen III. In the alternative pathway heme is generated via precorrin-2 requiring a total of six consecutive enzymatic steps. In the classical pathway uroporphyrinogen III is converted into heme via coproporphyrinogen III in four reaction steps. (b) Heme biosynthesis gene clusters in *M. barkeri*. Genes encoding enzymes which catalyze the early steps during heme formation are shown in black. Genes which were proposed to be important for the last three steps of the alternative heme biosynthesis pathway are shown in grey [3]. The gene numbers for *M. barkeri* Fusaro (Mbar_) are given below the arrows, the original gene designations [3] are given above the arrows, and the newly suggested names for the genes required for the last three steps are indicated in the grey arrows [6]. (c) The last three steps of the alternative heme biosynthesis pathway. First, the two acetate side chains at positions C-12 and C-18 of siroheme are decarboxylated to methyl groups by AhbAB resulting in 12,18-didecarboxysiroheme. AhbC removes the acetate side chains at positions C-2 and C-7 to form iron-coproporphyrin III which is finally converted into heme by AhbD through the oxidative decarboxylation of the propionate side chains at positions C-3 and C-8 to the corresponding vinyl groups. The newly suggested enzyme names are given above the arrows [6], and the original enzyme designations are given below the arrows in parentheses [3].

In contrast, in the course of the alternative heme biosynthesis route UROGEN is methylated to yield precorrin-2 which is then further transformed into heme via the intermediates sirohydrochlorin, siroheme (SH), 12,18-didecarboxysiroheme (DDSH), and iron-coproporphyrin III (Fe-COPRO III) (Figure 1(a)) [6]. Whereas it was known for almost twenty years that the first step of the alternative route is the *S*-adenosyl-L-methionine (SAM) dependent methylation of UROGEN to precorrin-2 [7–9], the remaining steps of the alternative heme biosynthesis pathway were elucidated only recently [6]. The bioinformatic analysis of completely sequenced archaeal genomes revealed the presence of potential heme biosynthesis gene clusters consisting of the “early” *hem* genes required for the formation of UROGEN and several *nir*-like genes which encode proteins that are homologous to the enzymes involved in heme *d*_1_ biosynthesis in denitrifying bacteria (Figure 1(b)) [3]. Similar genes were also identified in the genomes of sulfate-reducing bacteria such as *Desulfovibrio vulgaris* Hildenborough or *Desulfovibrio desulfuricans* [6, 10]. Since most archaea and sulfate-reducing bacteria do not possess a cytochrome *cd*_1_ nitrite reductase and, thus, do not synthesize heme *d*_1_, it was speculated that the *nir*-like genes identified in the genomes of these organisms might be involved in heme biosynthesis. Subsequently, it was shown *in vitro* that the corresponding recombinant Nir-like proteins from *D. vulgaris* Hildenborough and *D. desulfuricans* were indeed able to catalyze the stepwise conversion of siroheme into heme, that is, the last steps of the alternative heme biosynthesis pathway (Figure 1(c)). According to their newly identified function during the alternative heme biosynthesis, these proteins were given the prefix Ahb [6].

It was shown that AhbA and AhbB together catalyzed the decarboxylation of the acetate side chains on rings C and D of SH to yield DDSH. The same reaction also occurs during heme *d*_1_ biosynthesis in denitrifying bacteria where it is catalyzed by the homologous proteins NirD, NirL, NirG, and NirH [6]. Next, AhbC was found to catalyze the removal of the acetate side chains on rings A and B of DDSH to give Fe-COPRO III. AhbC is homologous to NirJ involved in heme *d*_1_ biosynthesis. Finally, AhbD was shown to convert Fe-COPRO III into heme by catalyzing the oxidative decarboxylation of the two propionate side chains on rings A and B to the corresponding vinyl groups [6]. AhbD is also homologous to the heme *d*_1_ biosynthesis protein NirJ, however, to a lower degree than AhbC. As mentioned above, the enzymatic activities of the Ahb proteins were shown mainly for the recombinant enzymes from the sulfate-reducing bacteria *D. vulgaris* Hildenborough and *D. desulfuricans*.

As previously reported and based on bioinformatic analysis, the archaea also possess homologues of these Nir-like alternative heme biosynthesis proteins which were named Ahb-Nir at the time [3]. In this nomenclature, Ahb-NirD corresponds to AhbA, Ahb-NirH to AhbB, Ahb-NirJ2 to AhbC, and Ahb-NirJ1 to AhbD (Figure 1(b)). In order to avoid further confusion, we will adopt here the four letter nomenclature (AhbA, AhbB, AhbC, and AhbD) also for the archaeal Ahb-Nir proteins.

Although an equivalent functionality can be inferred for the Ahb proteins from both groups of organisms, that is, sulfate-reducing bacteria and archaea, based on amino acid sequence similarities, these predictions still require experimental proof. Especially, the catalytic functions of AhbC and AhbD should be verified experimentally before the respective enzyme names are assigned, since both proteins share similarity with NirJ and are also quite similar to each other. Therefore, in this study we investigated the catalytic functions of the Ahb proteins from the methanogenic archaeon *Methanosarcina barkeri* and established their roles for the novel heme biosynthesis pathway in this organism.

## 2. Materials and Methods

### 2.1. Chemicals

All chemicals and reagents were obtained from Sigma-Aldrich (Taufkirchen, Germany), Carl Roth (Karlsruhe, Germany), Gerbu (Heidelberg, Germany), Merck (Darmstadt, Germany). DNA polymerases, restriction enzymes, and PCR requisites were purchased from New England Biolabs (Frankfurt a.M., Germany). Oligonucleotide primers and plasmid-miniprep kits were purchased from Metabion (Martinsried, Germany). PCR purification and gel extraction kits were obtained from Qiagen (Hilden, Germany). Ni-NTA agarose was purchased from Macherey-Nagel (Düren, Germany). Porphyrins were purchased from Frontier Scientific (Newark, USA). The four *E. coli* codon-optimized genes *Mbar_A1459*, *_A1460*, *_A1458,* and _*A1793* from *M. barkeri* were synthesized by Life Technologies GmbH (Darmstadt, Germany).

### 2.2. Construction of Plasmids

For the construction of plasmid pETDuet*nirDLGHJEN* containing the genes* nirD*, *nirL*, *nirG*, *nirH*, *nirJ*, *nirE, *and* nirN* from *Pseudomonas aeruginosa *the DNA fragment containing all seven genes was amplified by PCR with the primers NirDLGHJEN_NdeI_fw (G GAG CGA CAT ATG GAC GAC CTC TCC) and NirDLGHJEN_KpnI_bw (GAC GGT ACC TCA GTG CGA GGT TCC) using plasmid pHAE2 [11] as the template. The amplified DNA fragment was digested with the restriction enzymes *Nde*I and *Kpn*I (underlined) and ligated into the correspondingly digested vector pETDuet-1 (Novagen, Darmstadt, Germany) to generate pETDuet*nirDLGHJEN*. For the construction of plasmid pETDuet*ahbAB *the synthetic, codon-optimized gene *Mbar_A1460* (encoding AhbB) was amplified by PCR using the primers 15NirH_Mba_fw (C GTA CAT ATG GAT AAA ACC GAT GTG AAA) and 16NirH_Mba_rev (C GTT CTC GAG TTA CAG ACG AAC ACC G). The amplified DNA fragment was digested with the restriction enzymes *Nde*I and *Xho*I (underlined) and ligated into correspondingly digested vector pETDuet-1 yielding plasmid pETDuet*ahbB*. The synthetic, codon-optimized gene *Mbar_A1459* (encoding AhbA) was amplified by PCR using the primers 13NirD_Mba_fw (TG CAG AAT TCG ATG ATC GAT ATT GAC AAC CTG) and 14NirD_Mba_rev (TAA CGC GGC CGC TTA ACG AAT ATC GAA). The amplified DNA fragment was digested with the restriction enzymes *Eco*RI and *Not*I and ligated into the correspondingly digested plasmid pETDuet*ahbB* yielding plasmid pETDuet*ahbAB*. The plasmid pET22b*ahbABCD* containing the codon-optimized genes *Mbar_A1459*, *_A1460*, *_A1458,* and _*A1793* (encoding AhbABCD) from *M. barkeri* was constructed by the “link and lock” method as described before [12] using pET22b (Novagen) as the vector system. The plasmid pET22b*ahbABCD* was modified with the QuikChange Site-Directed Mutagenesis Kit (Stratagene) to insert a stop codon (underlined) either in *ahbD* by using the primers nirJ1_Stop (CTG AAT TGT GTT CAT TGA CGT GGT GCA AGC ACC AG) and nirJ1_Stop_antisense (CTG GT GCT TGC ACC ACG TCA ATG AAC ACA ATT CAG) to generate pET22b*ahbABCD*_*t78a*_ (producing AhbABC) or in *ahbC* to generate pET22b*ahbABC*_*a76t*_ *D* (producing AhbABD) by using the primers nirJ2_Stop (GGT CGT GAT AGC AAA TAA CTG CCG AGC CAT CTG) and nirJ2_Stop_antisense (CAG ATG GCT CGG CAG TTA TTT GCT ATC ACG ACC) for blocking the expression of the respective gene. For the construction of plasmid pETDuet*ahbD *the codon-optimized gene *Mbar_A1458* (encoding AhbD) was PCR amplified using the primers NirJ1_Duet_*Mfe*I_fw (C AAT TGG ATG ATT GCC ATG ACC AAT G) and NirJ1_pGEX_*Xho*I_bw (G CTC GAG TTA TTT TTT ACC CGG AC) containing *Mfe*I and *Xho*I restriction sites (underlined) and first cloned into the pJET1.2/blunt cloning vector (Thermo Scientific) according to the manufacturer's instructions. *Mbar_A1458* was then cut from this plasmid with *Mfe*I and *Xho*I endonucleases and the resulting DNA fragment was ligated into the correspondingly digested vector pETDuet-1 (Novagen) generating pETDuet*ahbD*. Plasmid pBCM-CysG6 encoding the recombinant siroheme synthase CysG from *Salmonella typhimurium* was kindly provided by Dr. Robert Schnell (Karolinska Institute, Stockholm, Sweden) [13].

### 2.3. Bacterial Strains and Growth Conditions

*E. coli* DH10B was used as the host for cloning. For production of recombinant proteins the *E. coli* strain BL21(DE3) was used. For recombinant protein production the *E. coli* strains carrying the corresponding vectors were grown at 37°C in LB-medium (aerobic growth conditions) or LB-medium containing 20 mM NaNO_3_ (anaerobic growth conditions) supplemented with appropriate antibiotics. Protein production was induced by adding 500 *μ*M IPTG (isopropyl-*β*-D-thiogalactopyranosid) to the cultures as soon as an optical density at 578 nm of 0.5 for aerobically and 0.2 for anaerobically grown cultures was reached. Cultures of *E. coli* cells containing plasmid pBCM-CysG6 were induced for protein production with 0.01% (w/v) arabinose. After induction the cells were further cultivated at 25°C overnight before harvesting by centrifugation and storing the cell pellet at −20°C.

### 2.4. *In Vivo* Enzyme Activity Assay

*In vivo *activity assays were performed by cultivating *E. coli* BL21(DE3) cells containing either only pBCM-CysG6 (production of *Salmonella typhimurium* CysG) or pBCM-CysG6 in combination with pETDuet*nirDLGHJEN* (production of *P. aeruginosa* NirDLGH), pETDuet*ahbAB* (production of *M. barkeri* AhbAB), pET22b*ahbABCD* (production of *M. barkeri* AhbABCD), pET22b*ahbABCD*_*t78a*_ (production of *M. barkeri* AhbABC), or pET22b*ahbABC*_*a76t*_ *D* (production of *M. barkeri* AhbABD), respectively. The cells were grown anaerobically in 120 mL plasma bottles and induced for protein production as described above. After overnight cultivation the cells were harvested and either directly used for HPLC sample preparation or stored at −20°C.

### 2.5. Purification of Enzymes

Recombinant AhbAB from *M. barkeri* was produced aerobically in *E. coli* BL21(DE3) containing the plasmid pETDuet*ahbAB* and the protein complex was purified by IMAC (immobilized metal ion affinity chromatography). The cell pellet was resuspended in buffer A (50 mM Tris/HCl, pH 7.5, 150 mM NaCl, 10% (v/v) glycerol) and the cells were disrupted using a French Press system (1000 psi). The soluble protein fraction was obtained by ultracentrifugation (45 min, 175000 ×g, 4°C). The supernatant was loaded on a Ni-NTA column and washed with 10 column volumes (CV) of buffer A and 2 CV of buffer A containing 50 mM imidazole before eluting the bound proteins with 7 CV of buffer A containing 300 mM imidazole. The protein content of the elution fractions was analyzed by SDS-polyacrylamide gel electrophoresis and the AhbAB containing fractions were pooled. The final buffer exchange against buffer A was performed with a NAP-25 Sephadex column (Illustra NAP-25, GE Healthcare). The purified AhbAB was stored at −20°C.

Recombinant AhbD from *M. barkeri* was produced in aerobically growing *E. coli* BL21(DE3) containing the plasmid pETDuet*ahbD*. After cell harvest all procedures done for the purification of AhbD were performed under anaerobic conditions in an anaerobic chamber (Coy Laboratories, Grass Lake, MI, USA). For the resuspension of the cell pellet buffer B (50 mM Tris/HCl, pH 7.5, 250 mM NaCl, 0.3% Triton X-100, 2 mM DTT) was used. The soluble protein fraction was obtained as described above. The supernatant was loaded on a 5 mL Blue Sepharose column (HiTrap Blue HP, GE Healthcare) equilibrated with 50 mM Tris/HCl, pH 7.5, containing 5 mM DTT and after sample application the column was washed thoroughly with this buffer. The bound proteins were eluted using a linear gradient from 0 M NaCl to 1 M NaCl in 50 mM Tris/HCl, pH 7.5, containing 5 mM DTT. The AhbD containing fractions (elution at about 500 mM NaCl) were pooled and the buffer was exchanged against 50 mM Tris/HCl, pH 8.0, containing 5 mM DTT. Then, the protein solution was loaded on an anion exchange column (Mono Q 5/50 GL, GE Healthcare) equilibrated with 50 mM Tris/HCl, pH 8.0, containing 5 mM DTT and the column was washed with the same buffer. A linear gradient from 0 M NaCl to 0.5 M NaCl in 50 mM Tris/HCl, pH 8.0, containing 5 mM DTT was used for the elution of the bound proteins. The AhbD containing fractions were pooled and concentrated to 5.6 mg/mL. The purified AhbD was stored at 4°C.

### 2.6. Dialysis of AhbAB

100 *μ*L of purified AhbAB at a concentration of 7.8 mg/mL were dialyzed at 4°C overnight against 100 mL of buffer A using a Slide-A-Lyzer dialysis cassette (10k MWCO, Thermo Scientific, Waltham, MA USA). Then, the used buffer A was exchanged against 100 mL of fresh buffer A and the dialysis was continued at 4°C for another three hours.

### 2.7. Determination of Protein Concentration

For the determination of protein concentrations the Bradford Reagent (Sigma-Aldrich) was used according to the manufacturer's instructions with BSA as the protein standard.

### 2.8. *In Vitro* Iron-Sulfur Cluster Reconstitution

The *in vitro* reconstitution of iron-sulfur clusters was performed as previously described [14]. After reconstitution the excess of iron and sulfide was removed by centrifugation and subsequent passage of the protein solution through a NAP-25 column (GE Healthcare) which was used according to the manufacturer's instructions.

### 2.9. Determination of Iron and Sulfide Contents

The iron content of purified AhbD was determined according to [15] after denaturation of the protein with 1 M perchloric acid and using bathophenanthroline as the chelating reagent. The sulfide content was determined as previously described [16].

### 2.10. Molecular Mass Determination

The native molecular mass of purified proteins was estimated by gel permeation chromatography as previously described [3].

### 2.11. *In Vitro* Enzyme Activity Assay for AhbAB

The assay was performed under anaerobic conditions in a glove box (Coy Laboratories). The substrate siroheme was produced *in vivo* by the production of recombinant CysG in *E. coli* as described above. Then, a crude cell free extract was prepared under anaerobic conditions from the siroheme containing *E. coli* cells which was used directly as the substrate solution for the activity assay. The assay mixtures contained in a total volume of 250 *μ*L of assay buffer (50 mM Tris/HCl, pH 7.5, 300 mM NaCl, 5% (v/v) glycerol) a final concentration of 20 *μ*M purified AhbAB which was incubated prior to substrate addition with 0 *μ*M, 5 *μ*M, or 20 *μ*M hemin. Then, 75 *μ*L of the siroheme containing cell free extract was added to start the reaction. For the control reaction AhbAB and hemin were omitted from the mixture. The reactions were incubated at 37°C for 19 h. Then, the accumulated tetrapyrroles were extracted from the assay mixtures and analyzed by HPLC as described below.

### 2.12. *In Vitro* Enzyme Activity Assay for AhbD

The assay was performed under anaerobic conditions in a glove box (Coy Laboratories). A stock solution of 500 *μ*M iron-coproporphyrin III (Fe-COPRO III) was generated according to Chim et al. [17]. The enzyme activity assay mixture contained 20 *μ*M Fe-COPRO III, 500 *μ*M *S*-adenosyl-L-methionine (SAM), 1 mM sodium dithionite (DT), and 5 *μ*M purified, reconstituted AhbD in buffer B. The reaction mixtures were incubated for 6 h and 15 h at 17°C. To stop the enzymatic reaction 5% (v/v) of concentrated HCl was added to the mixture which was stored at −20°C until further use.

### 2.13. Tetrapyrrole Extraction and Preparation of HPLC Samples

For the extraction of the potential heme bound to purified AhbAB the protein solution was mixed 1 : 1 (v/v) with solution 1 (50 mM Tris/HCl, pH 8.0, and 2% Tween 80) and 5% (v/v) of concentrated HCl was added to denature the proteins. After shaking the mixture on a Vortex-Genie 2 mixer (Scientific Industries Inc., Bohemia, NY, USA) for two minutes the sample was centrifuged (10 min, 16100 ×g). Then, an acetone/HCl mixture (39 : 1 (v/v)) was added 1 : 1 (v/v) to the supernatant followed by another centrifugation step. The resulting supernatant containing the extracted tetrapyrroles was analyzed by HPLC.

For the preparation of HPLC samples of the *in vivo* enzyme activity assays the cell pellets were resuspended 1 : 4 (w/v) in solution 1. The cells were disrupted with glass beads in a cell disruptor (10 min, 3000 rpm) and centrifuged (10 min, 16100 ×g). Addition of 600 *μ*L acetone/HCl (39 : 1 (v/v)) to 200 *μ*L of the supernatant and vortexing for 10 minutes was used to extract the tetrapyrroles and precipitate the proteins. The samples were centrifuged (10 min, 16100 ×g) and the supernatant was concentrated in a speed vac (Eppendorf, Hamburg, Germany) to a final volume of 200 *μ*L before adding 5% (v/v) of concentrated HCl and centrifuging (10 min, 16100 ×g). The obtained supernatant was used for HPLC analysis.

For the preparation of HPLC samples of the *in vitro* enzyme activity assays the assay mixture was mixed 1 : 1 (v/v) with acetone/HCl (39 : 1 (v/v)) followed by vortexing and centrifugation (10 min, 16100 ×g). 5% (v/v) of concentrated HCl was added to the supernatant and centrifuged again to remove residual protein before injection into the HPLC system.

### 2.14. Preparation of HPLC Standards

Hemin or Fe-COPRO III was first dissolved in 150 *μ*L of acetone/HCl (39 : 1 (v/v)) and then 75 *μ*L of solution 1 was added. The obtained tetrapyrrole solutions were centrifuged (10 min, 16100 ×g) and the supernatant was used for HPLC analysis.

### 2.15. High Performance Liquid Chromatography of Tetrapyrroles

Tetrapyrrole extracts were analyzed using a JASCO HPLC 2000 series system (Jasco, Gross-Umstadt, Germany). The tetrapyrroles were detected by photometric diode array analysis at 200–650 nm and by fluorescence measurements using an excitation wavelength of 409 nm and an emission wavelength of 630 nm.

The chromatographic separation of samples (30 *μ*L injection volume) containing heme and Fe-COPRO III was performed on an Equisil BDS C18 column (Dr. Maisch HPLC GmbH, Ammerbuch-Entringen, Germany) with 5 *μ*m particle size and 250 mm × 4.6 mm column dimensions at 25°C. The tetrapyrroles were eluted at a flow rate of 0.5 mL/min using a gradient system based on previously published methods [18]. Solvent A consisted of 1 M ammonium acetate (pH 5.2) (analytical grade, Sigma-Aldrich), solvent B of methanol (HPLC grade, Sigma-Aldrich), and solvent C of acetonitrile (HPLC grade, Sigma-Aldrich). At the time of sample injection the mobile phase consisted of 60% solvent A, 30% solvent B, and 10% solvent C. Within 30 min the content of solvent B was increased to 75% with concomitant decrease of solvent A to 15% while solvent C was held constant at 10%. Then, the content of solvent B was increased to 90% within 5 min while solvent C was held constant at 10%. These conditions (90% B and 10% C) were held for 15 min before returning to the initial conditions.

The separation of samples (20 *μ*L injection volume) containing siroheme and 12,18-didecarboxysiroheme was performed on a ReproSil-Pur C18 AQ column (Dr. Maisch HPLC GmbH) with 5 *μ*m particle size and 150 mm × 2 mm column dimensions at 25°C and a flow rate of 0.2 mL/min. For the elution of the tetrapyrroles a gradient system was used with solvent A consisting of 0.01% (v/v) formic acid in water (analytical grade, Merck) and solvent B consisting of acetonitrile (HPLC grade, Sigma-Aldrich). The initial conditions of the mobile phase consisted of 95% solvent A and 5% solvent B, which was increased to 20% within 6 min. Then, the content of solvent B was increased to 30% within 19 min and finally increased to 100% within 5 min and held for 10 min before returning to the initial conditions.

### 2.16. HPLC-MS Analysis of Siroheme and 12,18-Didecarboxysiroheme

The HPLC separation of the tetrapyrroles was performed as described above. The masses of the eluting tetrapyrroles were measured by ESI-MS on an LTQ XL linear ion trap mass spectrometer (Thermo Fisher Scientific) in the positive ion mode.

### 2.17. UV-Visible Absorption Spectroscopy

UV-visible absorption spectra of purified proteins were recorded using either a V-600 or a V-650 spectrophotometer (Jasco, Gross-Umstadt, Germany).

### 2.18. N-Terminal Sequencing

The N-terminal sequence of the purified protein was determined by Edman degradation.

## 3. Results and Discussion

### 3.1. AhbA and AhbB from *Methanosarcina barkeri* Catalyze the Decarboxylation of Siroheme to 12,18-Didecarboxysiroheme

**Figure 2:**
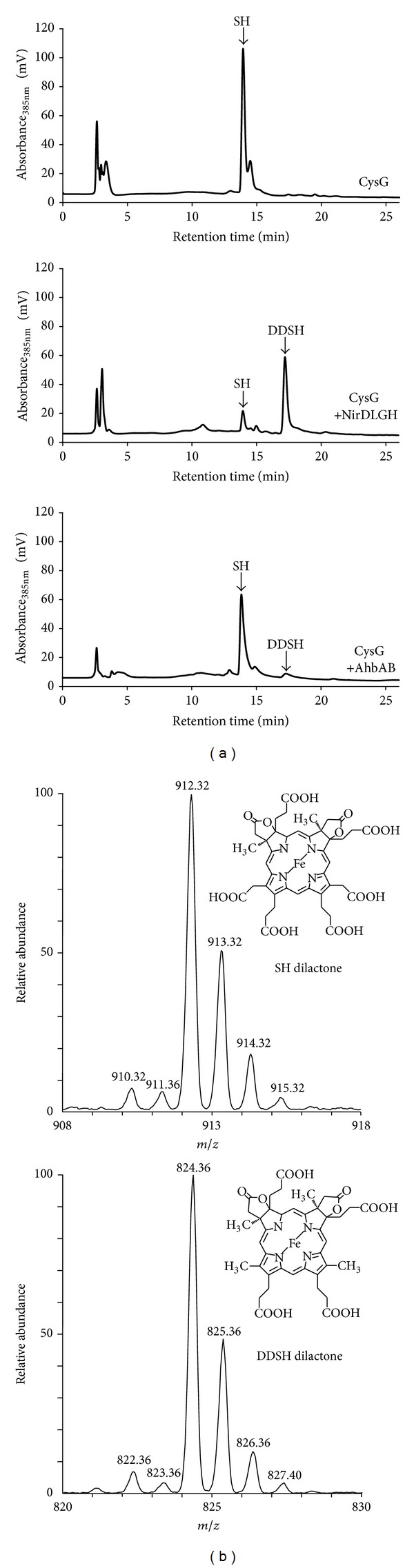
AhbA and AhbB from *M. barkeri* act as siroheme decarboxylase *in vivo*. (a) HPLC analysis of the tetrapyrroles accumulated in different *E. coli* strains. The HPLC analysis of cell free extracts from *E. coli* cells producing recombinant CysG (upper panel) shows the accumulation of siroheme (SH). In the presence of CysG and recombinant NirDLGH from *Pseudomonas aeruginosa *12,18-didecarboxysiroheme (DDSH) is formed (middle panel). In the presence of CysG and recombinant AhbAB from *M. barkeri* DDSH is formed (lower panel). (b) Isotopic mass spectra of SH (upper panel) and DDSH (lower panel) after HPLC separation. Both SH and DDSH eluted from the column in their dilactone form probably due to oxidation during tetrapyrrole extraction.

During the alternative heme biosynthesis route the acetate side chains on rings C and D of siroheme are decarboxylated to the corresponding methyl groups (Figure 1(c)). This reaction is catalyzed by enzymes (AhbA and AhbB) that are homologous to the heme *d*_1_ biosynthesis enzymes NirD, NirL, NirG, and NirH which catalyze the same reaction during heme *d*_1_ formation in denitrifying bacteria such as *Paracoccus pantotrophus* or *Pseudomonas aeruginosa* [6]. In *M. barkeri* the potential AhbA and AhbB proteins are encoded by the genes *Mbar_A1459* and *Mbar_A1460*, respectively (Figure 1(b)). Whereas in *M. barkeri* and other methanogenic archaea AhbA and AhbB are encoded by two distinct genes, these two genes are fused to one single gene in all other heme-synthesizing archaea [3]. Therefore, it is reasonable to assume that AhbA and AhbB together catalyze the decarboxylation of siroheme in *M. barkeri* as observed for the homologous proteins from *Desulfovibrio* species [6]. Thus, in the following we will name the siroheme decarboxylase AhbAB. In order to experimentally verify the predicted function of AhbAB from *M. barkeri* as a siroheme decarboxylase, we developed an *in vivo* enzyme activity assay. In this assay, siroheme, the substrate of AhbAB, was produced in *E. coli* by the recombinant siroheme synthase CysG from *Salmonella typhimurium* encoded on plasmid pBCM-CysG6 [13]. After overnight CysG production, the siroheme (SH) content of the *E. coli* cells was analyzed by tetrapyrrole extraction and HPLC analysis as described in Materials and Methods and shown in Figure 2(a). Next, we coproduced recombinant CysG together with recombinant AhbA and AhbB from *M. barkeri* in the same *E. coli* host and analyzed the accumulated tetrapyrroles after overnight protein production as before. Additionally, the same experiment was performed with recombinant NirD, NirL, NirG, and NirH from *P. aeruginosa*, constituting the potential siroheme decarboxylase for heme *d*_1_ formation in this organism, which were all coproduced with CysG as a positive control.

As shown in Figure 2(a), the HPLC analysis of the tetrapyrroles extracted from *E. coli* cells producing only CysG revealed the presence of one major compound at a retention time of 13.9 min which was identified as SH by HPLC-MS analysis (Figure 2(b)). The detected mass of 912.36 for the [M+H]^+^ ion corresponded to SH in its dilactone form. The formation of lactone derivatives of isobacteriochlorins was observed previously [19–21] and probably occurred during the tetrapyrrole extraction under aerobic conditions. Nevertheless, this result showed that SH, the potential substrate for AhbAB from *M. barkeri*, accumulated in CysG producing *E. coli* cells. When recombinant NirD, NirL, NirG, and NirH from *P. aeruginosa* were coproduced together with CysG in the same *E. coli* host, only residual amounts of SH were detected by HPLC analysis at a retention time of 13.9 min and a new major compound emerged at a retention time of about 17.2 min. The mass of this compound (824.36 for the [M+H]^+^ ion) corresponded to 12,18-didecarboxysiroheme (DDSH) in its dilactone form (Figure 2(b)). Thus, this result not only showed that our *in vivo* activity assay was suitable for the detection of SH decarboxylase activity, but also demonstrated for the first time that NirD, NirL, NirG, and NirH from *P. aeruginosa* indeed represent the SH decarboxylase for heme *d*_1_ formation in this denitrifying bacterium. Finally, DDSH was also formed *in vivo* when AhbA and AhbB from *M. barkeri* were co-produced together with CysG as judged by HPLC analysis of the extracted tetrapyrroles (Figure 2(a)). This result clearly established that the *M. barkeri* genes *Mbar_A1459* and *Mbar_A1460* encode the SH decarboxylase AhbAB.

### 3.2. AhbA and AhbB from *Methanosarcina barkeri* form a Heterodimeric, Heme-Binding Complex

**Figure 3:**
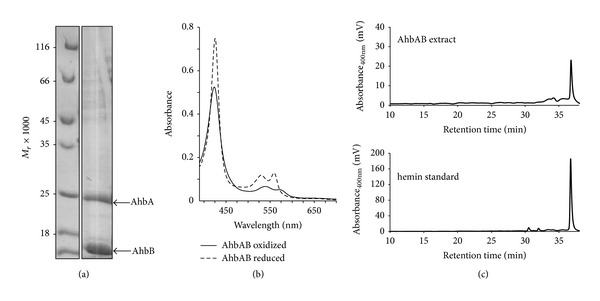
AhbAB from *M. barkeri* is a heme binding heterodimeric complex. (a) SDS-PAGE analysis of the purified AhbAB complex after copurification of His_6_-tagged AhbA with nontagged AhbB on Ni-NTA agarose. The *M*_*r*_ values of the marker proteins are indicated. (b) UV-visible absorption spectra of purified AhbAB in the oxidized form (as isolated, solid line) and the dithionite-reduced state (dashed line). (c) HPLC analysis of extracted tetrapyrroles of AhbAB. The comparison of the retention time of the extracted tetrapyrrole (upper panel) with a hemin standard (lower panel) suggests the presence of heme in purified AhbAB.

In *M. barkeri* and other methanogenic archaea, the SH decarboxylase AhbAB is composed of two protein subunits encoded by two distinct genes. In contrast, in all other heme-synthesizing archaea these two genes are fused to one single gene encoding the predicted SH decarboxylase in these organisms [3]. Therefore, we hypothesized that AhbA and AhbB from *M. barkeri* might form a mixed complex with each other *in vivo* which represents the physiological relevant form of the enzyme. In order to test this hypothesis, we coproduced recombinant AhbA carrying an N-terminal His_6_-tag together with nontagged AhbB in the same *E. coli* host. Then, His_6_-AhbA was purified by affinity chromatography on Ni-NTA agarose and the protein content of the final elution fraction was analyzed by SDS-PAGE. As shown in Figure 3(a), the nontagged AhbB was copurified together with His_6_-AhbA during this experiment strongly suggesting the formation of a mixed AhbAB complex *in vivo*. Further, the purified complex was analyzed by gel permeation chromatography which revealed the presence of a heterodimer (data not shown). Based on these novel results, we propose that the SH decarboxylase from *M. barkeri* is a heterodimeric enzyme consisting of the two subunits AhbA and AhbB.

Interestingly, the purified AhbAB complex was bright orange in color. The UV-visible absorption spectra of the purified protein in the as isolated and the dithionite-reduced form suggested the presence of a bound heme cofactor with absorption maxima at 423, 539, and 571 nm for the as isolated form and absorption maxima at 426, 530, and 559 nm in the dithionite-reduced form (Figure 3(b)). In order to characterize the bound chromophore in more detail, the purified protein was denatured by the addition of hydrochloric acid and the potential heme cofactor was extracted by acidified acetone. Subsequently, the extracted chromophore was analyzed by HPLC and was identified as heme *b* based on its retention time (Figure 3(c)) and UV-visible absorption spectrum (data not shown). Although the bound heme was removed from the AhbAB complex under denaturing conditions, we were unable to separate the protein-chromophore complex by dialysis. The ratio of heme to AhbAB was estimated by peak integration after HPLC analysis and was determined to be about 0.03 mol heme per mol AhbAB. In summary, these results clearly showed for the first time that AhbA and AhbB from *M. barkeri* form a heterodimeric AhbAB complex which partially contains a noncovalently, but tightly bound heme *b*.

The presence of heme *b* in the purified AhbAB complex was completely unexpected. Usually, heme *b* serves as a redox cofactor in enzymes or as a gas sensor in regulatory proteins. However, there is no need for a redox cofactor in the siroheme decarboxylase AhbAB and the homologous proteins NirDLGH which catalyze the same reaction during the biosynthesis of heme *d*_1_ do not contain any bound heme *b* after purification (K. Haufschildt and G. Layer, unpublished results). A potential role of the heme *b* as a cofactor for the catalytic activity of AhbAB seems unlikely considering that AhbAB catalyzes a reaction during the biosynthesis of the cofactor itself. Such a situation would raise the “chicken and egg” question. Alternatively, there are several different possibilities for the role of the bound heme in AhbAB. First, the presence of heme in the AhbAB preparation might reflect an end-product inhibition mechanism which controls the catalytic activity of AhbAB. In such a mechanism, the heme-free AhbAB complex would catalyze the decarboxylation of SH to DDSH in *M. barkeri* when heme is needed. As soon as the demand for heme is satisfied, the cofactor would bind to the AhbAB complex and might possibly inhibit its catalytic activity. Second, heme binding to AhbAB might control protein stability as observed for glutamyl-tRNA reductase which was reported to be inhibited upon heme binding and also seemed to be more amenable to proteolytic degradation in the heme-bound form [22–24]. The eukaryotic 5-aminolevulinic acid synthase (ALAS1) represents another example for a feedback-regulation by heme. In this case, gene expression and protein stability were reported to be dependent on the heme levels in mitochondria [25, 26]. Therefore, a third mechanism might be that heme-bound AhbAB might somehow influence gene expression of the heme biosynthesis genes. In this context it is interesting to note that the proteins AhbA and AhbB are annotated in the databases as transcriptional regulators belonging to the Lrp/AsnC-family.

### 3.3. Heme Does Not Inhibit the *In Vitro* Enzyme Activity of Purified AhbAB

**Figure 4:**
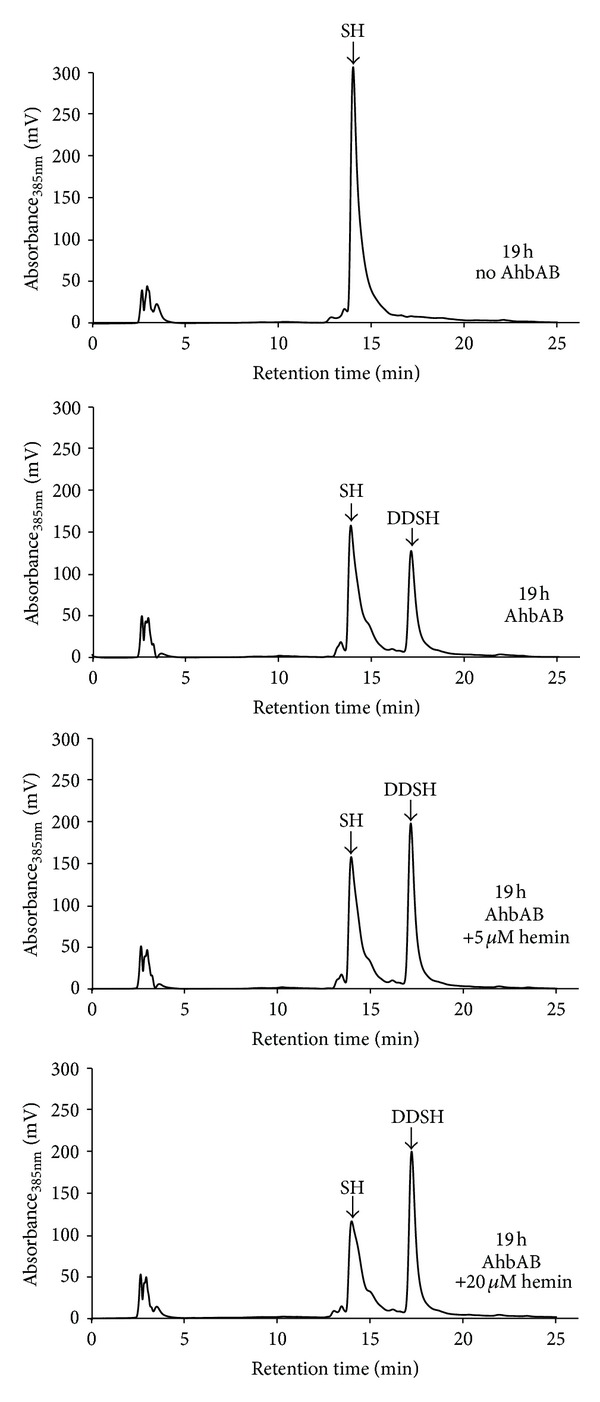
AhbAB is not inhibited by heme *in vitro*. The AhbAB enzyme activity assay was performed as described in Materials and Methods. In the absence of purified AhbAB no 12,18-didecarboxysiroheme (DDSH) is formed from siroheme (SH) (upper panel). In the presence of 20 *μ*M AhbAB, DDSH is formed independent of the amounts of added heme (other panels) suggesting that heme does not inhibit the *in vitro* activity of AhbAB.

In order to obtain more insights into the role of the heme bound to AhbAB we tested the possibility that heme might inhibit the catalytic activity of the protein. For this purpose, we tested the AhbAB activity in an *in vitro* enzyme activity assay in the absence and presence of different amounts of added heme as described in the Materials and Methods section. As shown in Figure 4, DDSH was formed by the action of purified AhbAB independent of the absence or presence of added heme. Even in the presence of equimolar amounts of heme with respect to the enzyme no inhibition of AhbAB was observed. These results indicate that heme does not inhibit the catalytic activity of AhbAB. Nevertheless, AhbAB might be subject to feedback-regulation by heme by one of the other possible mechanisms described above.

### 3.4. AhbC from *Methanosarcina barkeri* Catalyzes the Formation of Iron-Coproporphyrin III

During the alternative heme biosynthesis in archaea and sulfate-reducing bacteria the AhbC protein catalyzes the removal of the acetate side chains on rings A and B of DDSH yielding Fe-COPRO III. AhbC is a predicted Radical SAM enzyme homologous to the heme *d*_1_ biosynthesis enzyme NirJ [3, 6]. The heme biosynthesis gene clusters of *M. barkeri* (Figure 1(b)) contain two genes encoding two distinct NirJ-like proteins (previously named Ahb-NirJ1 and Ahb-NirJ2 [3]). The gene *Mbar_A1793* encodes a protein which shares 38.8% amino acid sequence identity with *P. aeruginosa* NirJ and, thus, most likely represents AhbC. The second NirJ-like protein in *M. barkeri*, which shares 29.5% sequence identity with *P. aeruginosa* NirJ, is encoded by the gene *Mbar_A1458* and probably represents AhbD, the terminal enzyme of the alternative heme biosynthesis pathway. In order to experimentally verify these functional predictions, we chose the same *in vivo* assay strategy as before for AhbAB. Thus, we coproduced recombinant CysG from *S. typhimurium* and AhbAB from *M. barkeri* together with either AhbC (Mbar_A1793) or AhbD (Mbar_A1458) or both AhbC and AhbD from *M. barkeri* in the same *E. coli* host. After overnight protein production the accumulated tetrapyrroles were extracted from the *E. coli* cell free extracts and analyzed by HPLC.

**Figure 5:**
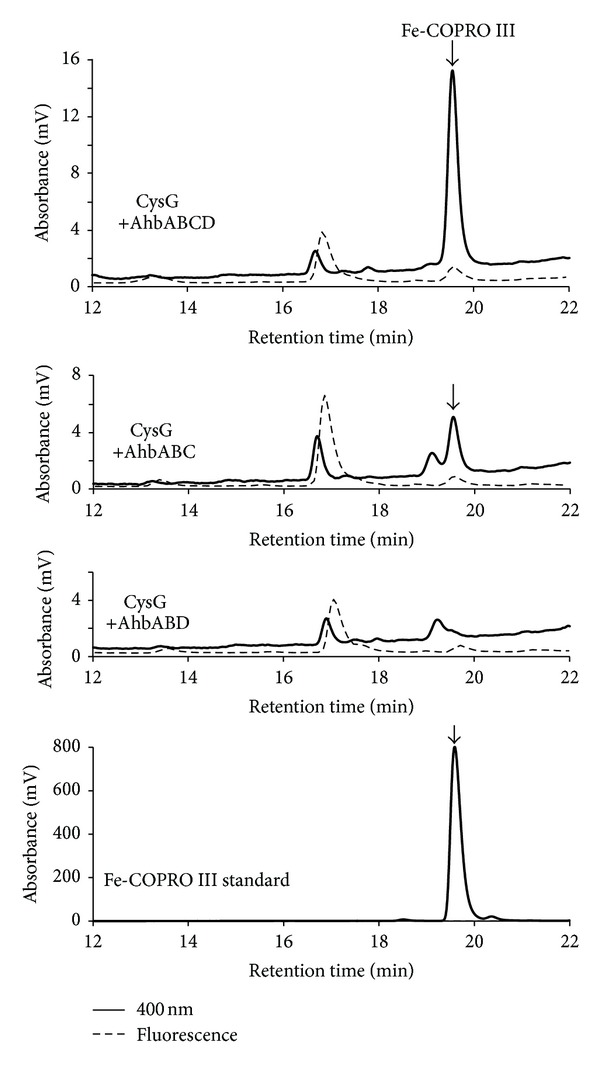
AhbC from *M. barkeri* generates iron-coproporphyrin III *in vivo*. The HPLC analysis of cell free extracts from *E. coli* cells producing recombinant CysG and recombinant AhbAB, AhbC, and AhbD from *M. barkeri* (upper panel) showed the accumulation of iron-coproporphyrin III (Fe-COPRO III). In *E. coli* cells producing recombinant CysG together with recombinant AhbAB and AhbC the formation of Fe-COPRO III was also observed (second panel). In contrast, the coproduction of CysG with AhbAB and AhbD did not result in the formation of Fe-COPRO III (third panel). The chromatographic behavior of the Fe-COPRO III standard is shown in the lower panel.

As shown in Figure 5, Fe-COPRO III (retention time 19.6 min) was produced in *E. coli* cells in the presence of recombinant CysG, AhbAB, and either both AhbC and AhbD or AhbC alone. In contrast, no Fe-COPRO III was produced in the presence of CysG, AhbAB, and AhbD when AhbC was missing in the *in vivo* assay. These results clearly demonstrated that the gene *Mbar_A1793* indeed encodes the enzyme AhbC which is responsible for the conversion of DDSH into Fe-COPRO III during the alternative heme biosynthesis in *M. barkeri*.

### 3.5. AhbD from *Methanosarcina barkeri* Is an Iron-Sulfur Cluster Containing Radical SAM Heme Synthase

**Figure 6:**
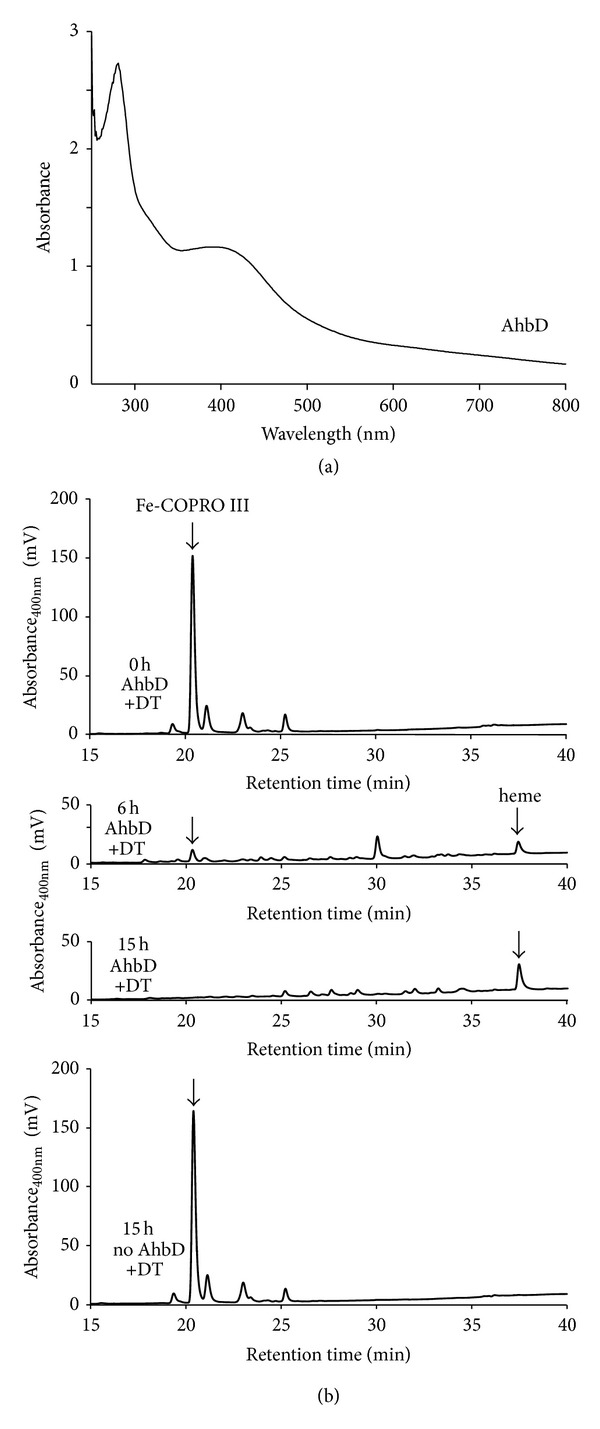
AhbD from *M. barkeri* acts as heme synthase *in vitro*. (a) UV-visible absorption spectrum of purified AhbD after *in vitro* iron-sulfur cluster reconstitution. (b) HPLC analysis of the tetrapyrrole content of AhbD enzyme activity assay mixtures. The *in vitro* activity assay mixture contained reconstituted AhbD (5 *μ*M), Fe-COPRO III (20 *μ*M), SAM (500 *μ*M), and sodium dithionite (1 mM) as reducing agent and was incubated at 17°C. The tetrapyrrole content of the mixture was analyzed by HPLC after 0 h (upper panel), 6 h (second panel), and 15 h (third panel) of incubation. After 6 h the formation of heme was detected (37.5 min) as well as the formation of a potential monovinyl intermediate (30.1 min). After 15 h of incubation the substrate Fe-COPRO III was completely consumed as well as the potential reaction intermediate and heme was detected as the sole reaction product. In contrast, no heme formation was observed after 15 h in a control reaction in which the purified AhbD was omitted from the assay mixture.

So far, we were able to assign the respective enzymatic functions to the *M. barkeri* proteins Mbar_A1459, Mbar_A1460, and Mbar_A1793 by the use of our *in vivo* activity assay. However, in order to establish the enzymatic function of Mbar_A1458 as a heme synthase that converts Fe-COPRO III into heme, the *in vivo* strategy in *E. coli* was not suitable, since *E. coli* produces heme by itself via the classical pathway. Therefore, the potential AhbD from *M. barkeri* (Mbar_A1458) was produced as a recombinant protein in *E. coli* and purified, in order to establish an *in vitro* enzyme activity assay. The recombinant AhbD did not carry any additional tag and was purified by affinity chromatography on a HiTrap Blue HP column (GE Healthcare) and subsequent anion exchange chromatography on a Mono Q 5/50 GL column (GE Healthcare) as described in the Materials and Methods section. Using this purification strategy about 0.9 mg of AhbD was obtained from 1 L of bacterial culture. After purification, the protein exhibited a slightly brownish color and the UV-visible absorption spectrum (not shown) of purified AhbD displayed a broad absorption feature at around 410 nm indicating the presence of an iron-sulfur cluster. As mentioned above, AhbD belongs to the family of Radical SAM enzymes and, therefore, the presence of a [4Fe-4S] cluster which is coordinated by the three cysteine residues of the characteristic amino acid sequence motif CX_3_CX_2_C was expected [27]. Moreover, the amino acid sequence of AhbD contains a second conserved cysteine-rich region at the C-terminus exhibiting the sequence motif CX_2_CX_5_CX_20_C. Therefore, AhbD might harbor a second iron-sulfur cluster in addition to the one that is coordinated by the cysteines of the Radical SAM cysteine motif (CX_3_CX_2_C) which is found at the N-terminus of AhbD. In order to obtain more insight into the iron-sulfur content of AhbD, we determined the iron content of the anaerobically purified protein. We observed that purified AhbD contained only 2.2 mol iron per mol AhbD which was less than expected for a protein containing at least one [4Fe-4S] cluster. However, this observation could be explained either by partial loss of the iron-sulfur cluster(s) during purification or by incomplete iron-sulfur cluster incorporation *in vivo* during recombinant protein production. Therefore, the purified AhbD was subjected to *in vitro* iron-sulfur cluster reconstitution as described in the Materials and Methods section. After cluster reconstitution and removal of excess iron and sulfide the protein exhibited an intense brown color and the UV-visible absorption spectrum indicated the presence of [4Fe-4S] clusters (Figure 6(a)). Subsequently, the iron and sulfide contents of the protein were determined. We observed that reconstituted AhbD contained 8.5 mol iron per mol AhbD and 5.1 mol sulfide per mol AhbD. These results indicate that AhbD most likely contains two [4Fe-4S] clusters and therefore belongs to the subfamily of Radical SAM enzymes that contain more than one iron-sulfur cluster [28].

In order to determine whether the purified, reconstituted AhbD was able to catalyze the conversion of Fe-COPRO III to heme, an *in vitro* enzyme activity assay was established. In this assay the purified protein was incubated with the substrate Fe-COPRO III together with the cofactor *S*-adenosyl-L-methionine (SAM) and sodium dithionite (DT) as the reductant for the iron-sulfur cluster of AhbD. After incubation of the mixture at 17°C for 0 h, 6 h, and 15 h the reaction was stopped by the addition of hydrochloric acid. The tetrapyrroles present in the reaction mixtures were extracted and subjected to HPLC analysis (Figure 6(b)). At the beginning of the reaction (0 h) only Fe-COPRO III was present in the mixture (Figure 6(b), first panel). After 6 h of incubation the amount of Fe-COPRO III had clearly decreased and two new compounds emerged at retention times of about 30.1 min and 37.5 min (Figure 6(b), second panel). The compound at 37.5 min retention time was heme as revealed by comparison with the chromatographic behavior of a hemin standard (see also Figure 3(c)). The compound at about 30.1 min retention time might be a monovinyl reaction intermediate. Finally, after 15 h the substrate Fe-COPRO III and the potential monovinyl intermediate completely disappeared and only the reaction product heme was detected in the reaction mixture (Figure 6(b), third panel). In contrast, when purified AhbD was omitted from the reaction mixture, no conversion of Fe-COPRO III to heme was observed after 15 h (Figure 6(b), fourth panel). The same was true when the “as isolated” enzyme (not reconstituted) was used (data not shown) showing that the full incorporation of the iron-sulfur clusters was required for AhbD activity. These results clearly established the enzymatic function of Mbar_A1458 as AhbD, that is, a heme synthase that converts Fe-COPRO III into heme during the alternative heme biosynthesis pathway in *Methanosarcina barkeri*.

## 4. Conclusion

In heme-synthesizing archaea heme is formed via an alternative pathway which differs significantly from the long known classical pathway found in most bacteria and eukaryotes. Previously, several archaeal genes were identified which were proposed to be involved in this alternative pathway, however, the function of only two of them was experimentally verified [3]. In this study, the predicted functions of the enzymes catalyzing the last three steps of the alternative heme biosynthesis pathway in *M. barkeri* were verified. Therefore, for *M. barkeri* the two enzymes which consecutively convert uroporphyrinogen III into sirohydrochlorin [3] and the three enzymes which convert siroheme into heme (this study) were biochemically characterized. The only missing enzyme to complete the whole pathway in this organism is the chelatase which incorporates iron into sirohydrochlorin to form siroheme. Several potential chelatases are encoded in the *M. barkeri* genome and future studies will have to show which of them acts as the iron-chelatase during heme formation.
